# Supplementary figures and images for: Interleukin-17, C-reactive protein, Neutrophil-to-Lymphocyte ratio, Lymphocyte-to-Monocyte ratio, and lipid profiles in healthy menopausal women with or without hot flashes: A cross-sectional study
Source: PLoS One. 2023 Nov 22;18(11):e0291804. doi: 10.1371/journal.pone.0291804 (PMC10664956; doi:10.1371/journal.pone.0291804)

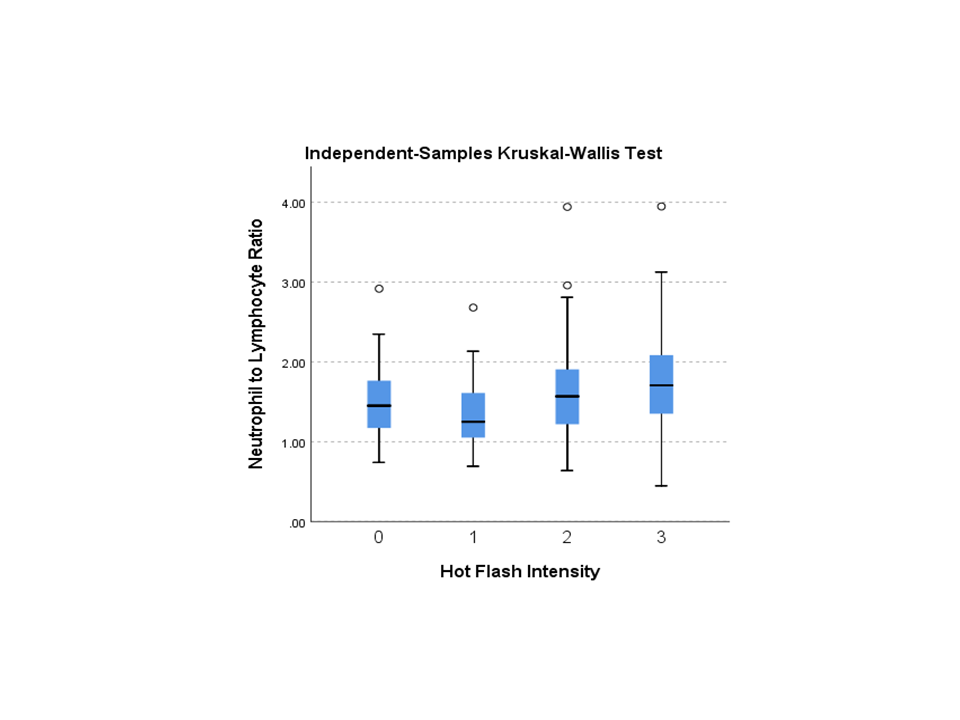

Supplement: S1 Fig — (TIF) [file pone.0291804.s001.tif]

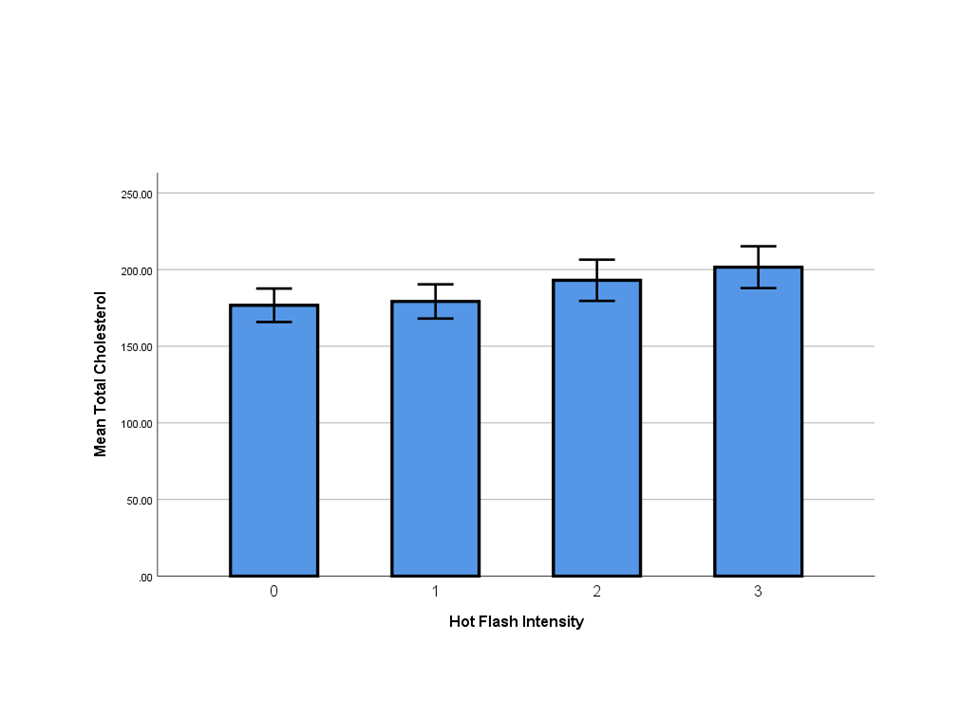

Supplement: S2 Fig — (TIF) [file pone.0291804.s002.tif]

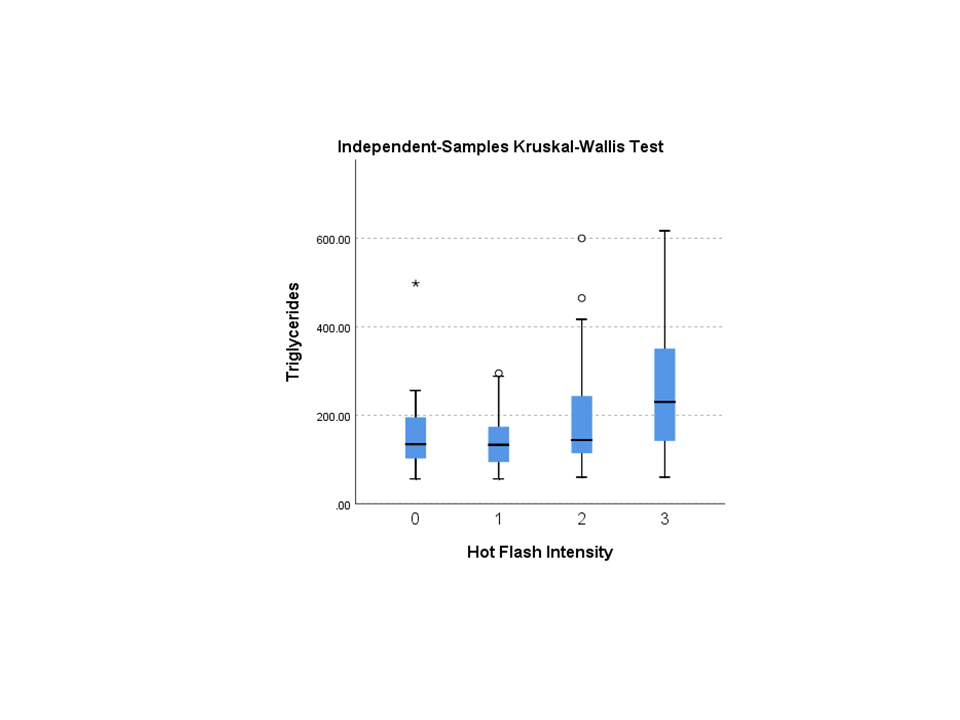

Supplement: S3 Fig — (TIF) [file pone.0291804.s003.tif]
