# Supplementary material for: Interleukin-17, C-reactive protein, Neutrophil-to-Lymphocyte ratio, Lymphocyte-to-Monocyte ratio, and lipid profiles in healthy menopausal women with or without hot flashes: A cross-sectional study
Source: PLoS One. 2023 Nov 22;18(11):e0291804. doi: 10.1371/journal.pone.0291804 (PMC10664956; doi:10.1371/journal.pone.0291804)
Supplement: S1 File — (PDF) [file pone.0291804.s004.pdf]

		Count	Column N %	Mean	Standard Deviation	Minimum	Maximum
age group	age <= 50	29	18.1%				
	age > 50	131	81.9%				
Age				54.64	4.26	40.00	60.00
Marital Status	married	121	75.6%				
	single	0	0.0%				
	widow	32	20.0%				
	divorced	7	4.4%				
Marriage Duration				32.32	9.08	2.00	49.00
Education	uneducated	23	14.4%				
	under diploma	95	59.4%				
	diploma	35	21.9%				
	graduate	7	4.4%				
Occupation	housewife	113	70.6%				
	worker	3	1.9%				
	farmer	11	6.9%				
	medical employee	13	8.1%				
	non medical employee	10	6.3%				
	free job	10	6.3%				
Spouse’s Age				58.65	6.46	38.00	79.00
Spouse’s Education	uneducated	9	7.4%				
	under diploma	77	63.6%				
	diploma	24	19.8%				
	graduate	11	9.1%				
Spouse’s Occupation	jobless	1	0.8%				
	worker	6	5.0%				
	farmer	11	9.1%				
	medical employee	1	0.8%				
	non medical employee	34	28.1%				
	free job	68	56.2%				
Place of residence	urban	132	82.5%				
	rural	28	17.5%				
Income Status	adequate	83	51.9%				
	inadequate	76	47.5%				
	over much	1	0.6%				
	Total	160	100.0%				

		Count	Column N %	Mean	Standard Deviation	Minimum	Maximum
Weight				68.66	6.96	50.00	86.00
Height				1.59	.05	1.46	1.70
Body Mass Index				27.01	2.38	18.14	30.09
BMI Status	Thin	1	0.6%				
	Normal	35	21.9%				
	Over Weight	121	75.6%				
	Obesity	3	1.9%				
Waist Circumference				96.86	8.40	73.00	117.00
Hip Circumference				108.22	7.66	90.00	126.00
WC/HC				.89	.04	.78	.98

		Count	Column N %	Mean	Standard Deviation	Minimum	Maximum
Physical Activities	no	77	48.1%				
	yes	83	51.9%				
Type of Physical Activity	walking	78	94.0%				
	aerobics	3	3.6%				
	mountain climbing	1	1.2%				
	yoga	1	1.2%				
Frequency of Physical Activites	twice a week	11	13.3%				
	three times a week	30	36.1%				
	everyday	42	50.6%				
Physical Activity Duration				47.89	19.60	20.00	120.00
Oily & Fast Food	no	118	73.8%				
	yes	42	26.3%				
Frequency of having Oily & Fast Food	never	118	73.8				
	once a month	30	18.8				
	twice a month	6	3.8				
	once a week	6	3.8				
Menopausal Age				48.66	3.73	35.00	58.00
Number of Gestation				3.83	1.83	.00	10.00
Number of Delivery				3.27	1.60	.00	8.00
Number of Children				3.17	1.54	.00	8.00

		Hot Flashes				P_value
		no		yes		
		Count	Column N %	Count	Column N %	
age group	age <= 50	4	10.0	25	20.8	*0.123
	age > 50	36	90.0	95	79.2	
	Total	40	100.0	120	100.0	
BMI Status	Thin	1	2.5	0	.0	**0.274
	Normal	10	25.0	25	20.8	
	Over Weight	28	70.0	93	77.5	
	Obesity	1	2.5	2	1.7	
	Total	40	100.0	120	100.0	
Marital Status	married	30	75.0	91	75.8	*0.223
	single	0	.0	0	.0	
	widow	10	25.0	22	18.3	
	divorced	0	.0	7	5.8	
	Total	40	100.0	120	100.0	
Education	uneducated	6	15.0	17	14.2	*0.985
	under diploma	24	60.0	71	59.2	
	diploma	8	20.0	27	22.5	
	graduate	2	5.0	5	4.2	
	Total	40	100.0	120	100.0	
Occupation	housewife	26	65.0	87	72.5	**0.174
	worker	1	2.5	2	1.7	
	farmer	2	5.0	9	7.5	
	medical employee	7	17.5	6	5.0	
	non medical employee	1	2.5	9	7.5	
	free job	3	7.5	7	5.8	
	Total	40	100.0	120	100.0	
Spouse's Education	uneducated	2	6.7	7	7.7	**0.933
	under diploma	21	70.0	56	61.5	
	diploma	5	16.7	19	20.9	
	graduate	2	6.7	9	9.9	
	Total	30	100.0	91	100.0	
Spouse's Occupation	jobless	0	.0	1	1.1	**0.611
	worker	1	3.3	5	5.5	
	farmer	5	16.7	6	6.6	
	medical employee	0	.0	1	1.1	
	non medical employee	9	30.0	25	27.5	
	free job	15	50.0	53	58.2	

	Total	30	100.0	91	100.0	
Place of residence	urban	33	82.5	99	82.5	*0.999
	rural	7	17.5	21	17.5	
	Total	40	100.0	120	100.0	
Income Status	adequate	18	45.0	65	54.2	**0.190
	inadequate	21	52.5	55	45.8	
	over much	1	2.5	0	.0	
	Total	40	100.0	120	100.0	
Physical Activities	no	18	45.0	59	49.2	*0.648
	yes	22	55.0	61	50.8	
	Total	40	100.0	120	100.0	
Type of Physical Activity	walking	20	90.9	58	95.1	*0.558
	aerobics	2	9.1	1	1.6	
	mountain climbing	0	.0	1	1.6	
	yoga	0	.0	1	1.6	
	Total	22	100.0	61	100.0	
Frequency of Physical Activites	twice a week	2	9.1	9	14.8	*0.615
	three times a week	7	31.8	23	37.7	
	everyday	13	59.1	29	47.5	
	Total	22	100.0	61	100.0	
Oily & Fast Food	no	28	70.0	90	75.0	*0.534
	yes	12	30.0	30	25.0	
	Total	40	100.0	120	100.0	
Frequency of having Oily & Fast Food	never	28	70.0	90	75.0	*0.440
	once a month	7	17.5	23	19.2	
	twice a month	3	7.5	3	2.5	
	once a week	2	5.0	4	3.3	
	Total	40	100.0	120	100.0	

*Pearson Chi-Square Test

**Fisher's Exact Test

Tests of Normality							
	Hot Flashes	Kolmogorov-Smirnov[a]			Shapiro-Wilk		
		Statistic	df	Sig.	Statistic	df	Sig.
Menopausal Age	no	.153	40	.020	.953	40	.095
	yes	.099	120	.006	.978	120	.051
Elapsed time since the last menstruation	no	.163	40	.009	.911	40	.004
	yes	.148	120	.000	.888	120	.000
Mean Hot Flashes	no	.	40	.	.	40	.
	yes	.177	120	.000	.870	120	.000
Elapsed time since the onset of hot flashes	no	.	40	.	.	40	.
	yes	.149	120	.000	.893	120	.000
a. Lilliefors Significance Correction							

		Hot Flashes		
		no	yes	*P_Value
Age	Mean	55.65	54.30	0.082
	Standard Deviation	3.63	4.41	
	Median	56.00	54.00	
Body Mass Index	Mean	26.63	27.14	0.319
	Standard Deviation	2.93	2.16	
	Median	27.47	27.57	
WC/HC	Mean	.89	.90	0.583
	Standard Deviation	.04	.03	
	Median	.89	.90	
Marriage Duration	Mean	34.73	31.52	0.053
	Standard Deviation	8.81	9.06	
	Median	37.00	33.00	
Spouse’s Age	Mean	60.00	58.21	0.189
	Standard Deviation	6.46	6.43	
	Median	60.00	59.00	
Physical Activity Duration	Mean	42.05	50.00	0.103
	Standard Deviation	14.53	20.84	
	Median	45.00	60.00	

*Independent Samples T-Test

		Hot Flashes			P*
		no	yes	Total	
Menopausal Age	Mean	49.35	48.43	48.66	0.216
	Standard Deviation	3.03	3.92	3.73	
	Median	49.50	49.00	49.00	
	Percentile 25	48.00	46.00	47.00	
	Percentile 75	52.00	51.00	51.00	
Elapsed time since the last menstruation	Mean	76.20	70.40	71.85	0.254
	Standard Deviation	43.86	50.74	49.04	
	Median	72.00	60.00	60.00	
	Percentile 25	36.00	24.00	36.00	
	Percentile 75	96.00	96.00	96.00	
Mean Hot Flashes	Mean	.00	5.90	4.42	-
	Standard Deviation	.00	3.76	4.14	
	Median	.00	5.35	2.28	
	Percentile 25	.00	2.00	.46	
	Percentile 75	.00	10.07	8.46	
Elapsed time since the onset of hot flashes	Mean	.00	72.27	54.20	-
	Standard Deviation	.00	50.17	53.56	
	Median	.00	60.00	48.00	
	Percentile 25	.00	36.00	6.00	
	Percentile 75	.00	96.00	84.00	

- *Mann Whitney U Test

		Hot Flashes Intensity								
		1		2		3		Total		
		Count	Column N %	Count	Column N %	Count	Column N %	Count	Column N %	P
age group	age <= 50	3	7.1%	10	23.3%	12	34.3%	25	20.8%	
	age > 50	39	92.9%	33	76.7%	23	65.7%	95	79.2%	
BMI Status	Thin	0	0.0%	0	0.0%	0	0.0%	0	0.0%	
	Normal	9	21.4%	13	30.2%	3	8.6%	25	20.8%	
	Over Weight	32	76.2%	30	69.8%	31	88.6%	93	77.5%	
	Obesity	1	2.4%	0	0.0%	1	2.9%	2	1.7%	
Marital Status	married	34	81.0%	28	65.1%	29	82.9%	91	75.8%	
	single	0	0.0%	0	0.0%	0	0.0%	0	0.0%	
	widow	6	14.3%	12	27.9%	4	11.4%	22	18.3%	
	divorced	2	4.8%	3	7.0%	2	5.7%	7	5.8%	
Education	uneducated	2	4.8%	10	23.3%	5	14.3%	17	14.2%	
	under diploma	28	66.7%	21	48.8%	22	62.9%	71	59.2%	
	diploma	11	26.2%	10	23.3%	6	17.1%	27	22.5%	
	graduate	1	2.4%	2	4.7%	2	5.7%	5	4.2%	
Occupation	housewife	33	78.6%	26	60.5%	28	80.0%	87	72.5%	
	worker	0	0.0%	2	4.7%	0	0.0%	2	1.7%	
	farmer	2	4.8%	4	9.3%	3	8.6%	9	7.5%	
	medical employee	1	2.4%	2	4.7%	3	8.6%	6	5.0%	
	non medical employee	3	7.1%	5	11.6%	1	2.9%	9	7.5%	
	free job	3	7.1%	4	9.3%	0	0.0%	7	5.8%	
Spouse's Education	uneducated	2	5.9%	2	7.1%	3	10.3%	7	7.7%	
	under diploma	22	64.7%	19	67.9%	15	51.7%	56	61.5%	
	diploma	5	14.7%	7	25.0%	7	24.1%	19	20.9%	
	graduate	5	14.7%	0	0.0%	4	13.8%	9	9.9%	
Spouse's Occupation	jobless	0	0.0%	0	0.0%	1	3.4%	1	1.1%	
	worker	1	2.9%	2	7.1%	2	6.9%	5	5.5%	
	farmer	1	2.9%	2	7.1%	3	10.3%	6	6.6%	
	medical employee	0	0.0%	0	0.0%	1	3.4%	1	1.1%	
	non medical employee	9	26.5%	9	32.1%	7	24.1%	25	27.5%	
	free job	23	67.6%	15	53.6%	15	51.7%	53	58.2%	
Place of residence	urban	36	85.7%	35	81.4%	28	80.0%	99	82.5%	
	rural	6	14.3%	8	18.6%	7	20.0%	21	17.5%	
Income Status	adequate	23	54.8%	25	58.1%	17	48.6%	65	54.2%	

	inadequate	19	45.2%	18	41.9%	18	51.4%	55	45.8%	
	over much	0	0.0%	0	0.0%	0	0.0%	0	0.0%	
Physical Activities	no	18	42.9%	23	53.5%	18	51.4%	59	49.2%	
	yes	24	57.1%	20	46.5%	17	48.6%	61	50.8%	
Type of Physical Activity	walking	23	95.8%	19	95.0%	16	94.1%	58	95.1%	
	aerobics	0	0.0%	0	0.0%	1	5.9%	1	1.6%	
	mountain climbing	1	4.2%	0	0.0%	0	0.0%	1	1.6%	
	yoga	0	0.0%	1	5.0%	0	0.0%	1	1.6%	
Frequency of Physical Activities	twice a week	5	20.8%	3	15.0%	1	5.9%	9	14.8%	
	three times a week	9	37.5%	10	50.0%	4	23.5%	23	37.7%	
	everyday	10	41.7%	7	35.0%	12	70.6%	29	47.5%	
Oily & Fast Food	no	34	81.0%	33	76.7%	23	65.7%	90	75.0%	
	yes	8	19.0%	10	23.3%	12	34.3%	30	25.0%	
Frequency of having Oily & Fast Food	once a month	6	75.0%	8	80.0%	9	75.0%	23	76.7%	
	twice a month	0	0.0%	1	10.0%	2	16.7%	3	10.0%	
	once a week	2	25.0%	1	10.0%	1	8.3%	4	13.3%	

Pearson Chi-Square Tests

		Hot Flashes Intensity
age group	Chi-square	8.766
	df	2
	Sig.	.012
BMI Status	Chi-square	6.394
	df	4
	Sig.	.172[c]
Marital Status	Chi-square	4.639
	df	4
	Sig.	.326
Education	Chi-square	7.644
	df	6
	Sig.	.265

Occupation	Chi-square	12.107
	df	10
	Sig.	.278[c]
Spouse's Education	Chi-square	6.121
	df	6
	Sig.	.410
Spouse's Occupation	Chi-square	7.426
	df	10
	Sig.	.685[c]
Place of residence	Chi-square	.488
	df	2
	Sig.	.783
Income Status	Chi-square	.721
	df	2
	Sig.	.697
Physical Activities	Chi-square	1.062
	df	2
	Sig.	.588
Type of Physical Activity	Chi-square	6.183
	df	6
	Sig.	.403[c]
Frequency of Physical Activities	Chi-square	5.952
	df	4
	Sig.	.203
Oily & Fast Food	Chi-square	2.473
	df	2
	Sig.	.290
Frequency of having Oily & Fast Food	Chi-square	2.480
	df	4
	Sig.	.648[c]
c. The minimum expected cell count in this subtable is less than one. Chi-square results may be invalid.		

Tests of Normality

	Hot Flashes Intensity	Kolmogorov-Smirnov[a]			Shapiro-Wilk		
		Statistic	df	Sig.	Statistic	df	Sig.
Menopausal Age	0	.153	40	.020	.953	40	.095
	1	.144	42	.028	.959	42	.138
	2	.094	43	.200*	.977	43	.528
	3	.108	35	.200*	.962	35	.269
Elapsed time since the last menstruation	0	.163	40	.009	.911	40	.004
	1	.166	42	.005	.918	42	.005
	2	.138	43	.038	.905	43	.002
	3	.131	35	.134	.917	35	.012
Mean Hot Flashes	0	.	40	.	.	40	.
	1	.115	42	.183	.977	42	.531
	2	.123	43	.098	.955	43	.092
	3	.150	35	.044	.927	35	.023
Elapsed time since the onset of hot flashes	0	.	40	.	.	40	.
	1	.174	42	.003	.917	42	.005
	2	.133	43	.054	.910	43	.003
	3	.149	35	.048	.935	35	.039

*. This is a lower bound of the true significance.

a. Lilliefors Significance Correction

		Hot Flashes Intensity				P*
		1	2	3	Total	
Menopausal Age	Mean	48.60	47.95	48.83	48.43	0.430
	Standard Deviation	4.57	3.63	3.43	3.92	
	Median	50.00	48.00	49.00	49.00	
	Percentile 25	45.00	46.00	46.00	46.00	
	Percentile 75	51.00	50.00	52.00	51.00	
Elapsed time since the last menstruation	Mean	87.14	77.30	41.83	70.40	<0.001
	Standard Deviation	54.45	53.62	25.27	50.74	
	Median	84.00	60.00	36.00	60.00	
	Percentile 25	48.00	24.00	24.00	24.00	
	Percentile 75	108.00	108.00	60.00	96.00	
Mean Hot Flashes	Mean	1.73	6.09	10.67	5.90	<0.001
	Standard Deviation	.34	1.83	.52	3.76	
	Median	1.75	5.57	10.64	5.35	
	Percentile 25	1.50	4.64	10.21	2.00	
	Percentile 75	2.07	7.57	10.92	10.07	
Elapsed time since the onset of hot flashes	Mean	88.19	78.42	45.60	72.27	<0.001
	Standard Deviation	53.70	53.75	25.88	50.17	
	Median	84.00	72.00	48.00	60.00	
	Percentile 25	48.00	30.00	24.00	36.00	
	Percentile 75	108.00	108.00	66.00	96.00	

- *Kruskal Wallis

Descriptives					
		N	Mean	Std. Deviation	P
Age	1	42	55.8571	4.22288	0.002
	2	43	54.3953	4.56247	
	3	35	52.3143	3.70033	
	Total	120	54.3000	4.40702	
Body Mass Index	1	42	27.2792	2.12339	0.07
	2	43	26.5692	2.31281	
	3	35	27.6730	1.90534	
	Total	120	27.1396	2.16488	
WC/HC	1	42	.8927	.04110	0.411
	2	43	.8931	.03391	
	3	35	.9023	.02726	
	Total	120	.8957	.03491	
Marriage Duration	1	42	32.1190	8.57735	0.829
	2	43	30.9070	9.45368	
	3	35	31.5429	9.34421	
	Total	120	31.5167	9.06094	
Spouse’s Age	1	34	59.3235	7.62262	0.086
	2	28	59.1071	5.43200	
	3	29	56.0345	5.37509	
	Total	91	58.2088	6.43345	
Physical Activity Duration	1	24	47.0833	21.26012	0.576
	2	20	53.7500	20.44730	
	3	17	49.7059	21.24784	
	Total	61	50.0000	20.83667	

Tests of Normality

	Hot Flashes	Kolmogorov-Smirnov[a]			Shapiro-Wilk		
		Statistic	df	Sig.	Statistic	df	Sig.
White Blood Cells	no	.164	40	.009	.896	40	.001
	yes	.092	120	.014	.974	120	.019
Neutrophil Percentage	no	.148	40	.028	.962	40	.196
	yes	.080	120	.059	.980	120	.077
Lymphocyte Percentage	no	.108	40	.200*	.983	40	.786
	yes	.067	120	.200*	.987	120	.289
Monocyte Percentage	no	.119	40	.161	.968	40	.319
	yes	.111	120	.001	.952	120	.000
Neutrophil Count	no	.148	40	.028	.951	40	.085
	yes	.081	120	.052	.947	120	.000
Lymphocyte Count	no	.139	40	.049	.910	40	.004
	yes	.091	120	.016	.924	120	.000
Monocyte Count	no	.166	40	.007	.901	40	.002
	yes	.079	120	.063	.980	120	.065
Neutrophil to Lymphocyte Ratio	no	.101	40	.200*	.959	40	.152
	yes	.098	120	.007	.907	120	.000
Lymphocyte to Monocyte Ratio	no	.248	40	.000	.685	40	.000
	yes	.194	120	.000	.629	120	.000
Total Cholesterol	no	.127	40	.106	.963	40	.213
	yes	.070	120	.200*	.984	120	.163
Triglycerides	no	.121	40	.146	.813	40	.000
	yes	.172	120	.000	.851	120	.000
HDL-Cholesterol	no	.144	40	.035	.933	40	.019
	yes	.085	120	.034	.976	120	.030
LDL-Cholesterol	no	.127	40	.106	.958	40	.142
	yes	.066	120	.200*	.979	120	.058
TC/HDL-Cholesterol	no	.110	40	.200*	.980	40	.693
	yes	.132	120	.000	.760	120	.000
Estradiol	no	.205	40	.000	.782	40	.000
	yes	.180	120	.000	.848	120	.000
IL-17	no	.204	40	.000	.854	40	.000
	yes	.088	120	.024	.942	120	.000
high sensitivity CRP	no	.150	40	.023	.925	40	.011
	yes	.193	120	.000	.772	120	.000

a. Lilliefors Significance Correction

*. This is a lower bound of the true significance.

	Hot Flashes										P
	no					yes					
	Mean	SD	Median	P25	P75	Mean	SD	Median	P25	P75	
White Blood Cells	6.67	1.45	6.20	5.60	7.25	6.74	1.63	6.40	5.65	7.70	0.669
Neutrophil Percentage	.54	.08	.55	.48	.60	.55	.08	.55	.49	.60	0.658
Lymphocyte Percentage	.38	.07	.37	.34	.42	.37	.08	.38	.32	.43	0.728
Monocyte Percentage	.05	.02	.05	.04	.06	.05	.02	.05	.03	.06	0.141
Neutrophil Count	3.60	1.01	3.41	2.87	4.24	3.69	1.13	3.54	2.91	4.30	0.765
Lymphocyte Count	2.49	.60	2.32	2.10	2.75	2.51	.80	2.42	2.00	2.88	0.912
Monocyte Count	.35	.17	.30	.26	.42	.31	.13	.29	.21	.40	0.263
Neutrophil to Lymphocyte Ratio	1.50	.48	1.45	1.17	1.76	1.57	.58	1.47	1.16	1.89	0.610
Lymphocyte to Monocyte Ratio	8.72	5.49	7.27	5.79	9.13	9.92	7.36	8.00	6.00	11.42	0.278
Total Cholesterol	176.70	34.13	183.50	153.00	199.50	190.66	40.62	185.00	162.50	222.50	0.095
Triglycerides	152.50	78.15	134.50	102.00	195.50	195.57	120.11	149.50	111.50	238.00	0.072
HDL-Cholesterol	40.40	5.87	39.00	36.00	44.50	42.61	8.69	42.00	37.00	47.50	0.136
LDL-Cholesterol	103.03	27.82	108.50	78.50	128.50	112.12	39.68	109.00	83.00	139.50	0.328
TC/HDL-Cholesterol	4.43	.95	4.37	3.74	5.17	4.67	1.62	4.28	3.68	5.18	0.917
Estradiol	10.91	10.20	8.30	4.15	12.70	11.05	9.17	7.90	4.45	16.00	0.757
IL-17	51.94	26.57	41.21	33.00	66.19	53.25	30.58	47.38	29.91	75.35	0.830
high sensitivity CRP	2.99	1.46	3.00	2.00	5.50	2.89	1.87	2.75	2.00	6.00	0.351

Tests of Normality

	Hot Flashes Intensity	Kolmogorov-Smirnov[a]			Shapiro-Wilk		
		Statistic	df	Sig.	Statistic	df	Sig.
White Blood Cells	1	.083	42	.200*	.967	42	.254
	2	.148	43	.018	.945	43	.041
	3	.109	35	.200*	.970	35	.436
Neutrophil Percentage	1	.070	42	.200*	.990	42	.966
	2	.105	43	.200*	.948	43	.051
	3	.125	35	.186	.949	35	.102
Lymphocyte Percentage	1	.078	42	.200*	.987	42	.914
	2	.097	43	.200*	.984	43	.815
	3	.118	35	.200*	.934	35	.037
Monocyte Percentage	1	.151	42	.018	.920	42	.006
	2	.164	43	.005	.944	43	.036
	3	.123	35	.200*	.960	35	.234
Neutrophil Count	1	.108	42	.200*	.972	42	.388
	2	.149	43	.018	.918	43	.005
	3	.122	35	.200*	.905	35	.005
Lymphocyte Count	1	.109	42	.200*	.920	42	.006
	2	.088	43	.200*	.964	43	.191
	3	.143	35	.069	.858	35	.000
Monocyte Count	1	.129	42	.075	.951	42	.068
	2	.081	43	.200*	.984	43	.786
	3	.154	35	.035	.936	35	.043
Neutrophil to Lymphocyte Ratio	1	.134	42	.056	.942	42	.035
	2	.121	43	.124	.906	43	.002
	3	.138	35	.092	.916	35	.011
Lymphocyte to Monocyte Ratio	1	.166	42	.005	.906	42	.002
	2	.209	43	.000	.690	43	.000
	3	.294	35	.000	.523	35	.000
Total Cholesterol	1	.112	42	.200*	.969	42	.313
	2	.105	43	.200*	.983	43	.758
	3	.115	35	.200*	.959	35	.211
Triglycerides	1	.116	42	.181	.920	42	.006
	2	.220	43	.000	.826	43	.000
	3	.135	35	.108	.937	35	.045
HDL-Cholesterol	1	.064	42	.200*	.987	42	.920

	2	.169	43	.004	.942	43	.031
	3	.106	35	.200*	.971	35	.467
LDL-Cholesterol	1	.094	42	.200*	.975	42	.487
	2	.070	43	.200*	.974	43	.446
	3	.092	35	.200*	.932	35	.033
TC/HDL-Cholesterol	1	.161	42	.008	.815	42	.000
	2	.126	43	.086	.905	43	.002
	3	.184	35	.004	.689	35	.000
Estradiol	1	.156	42	.012	.847	42	.000
	2	.238	43	.000	.855	43	.000
	3	.186	35	.003	.838	35	.000
IL-17	1	.089	42	.200*	.913	42	.004
	2	.124	43	.095	.939	43	.025
	3	.141	35	.075	.944	35	.074
high sensitivity CRP	1	.221	42	.000	.891	42	.001
	2	.213	43	.000	.839	43	.000
	3	.277	35	.000	.687	35	.000

*. This is a lower bound of the true significance.

a. Lilliefors Significance Correction

	Hot Flashes Intensity									P
	1			2			3			
	Mean	SD	Median	Mean	SD	Median	Mean	SD	Median	
White Blood Cells	6.33	1.62	6.25	6.87	1.62	6.40	7.08	1.61	7.20	0.210
Neutrophil Percentage	.52	.07	.52	.55	.08	.56	.57	.09	.58	0.011
Lymphocyte Percentage	.40	.07	.40	.36	.08	.35	.36	.08	.35	0.034
Monocyte Percentage	.05	.02	.05	.05	.02	.05	.04	.02	.04	0.257
Neutrophil Count	3.27	.96	3.21	3.80	1.09	3.84	4.07	1.24	3.94	0.023
Lymphocyte Count	2.53	.81	2.51	2.46	.76	2.38	2.55	.86	2.36	0.973
Monocyte Count	.30	.12	.28	.32	.13	.32	.30	.14	.27	0.473
Neutrophil to Lymphocyte Ratio	1.36	.41	1.25	1.65	.62	1.57	1.72	.65	1.71	0.013
Lymphocyte to Monocyte Ratio	9.71	4.78	8.29	9.31	6.32	7.00	10.93	10.54	8.25	0.457
Total Cholesterol	179.19	35.80	176.50	193.00	43.71	191.00	201.54	39.72	207.00	0.033
Triglycerides	140.79	60.84	133.00	193.81	118.13	144.00	263.46	142.59	230.00	0.001
HDL-Cholesterol	43.07	8.53	43.00	42.33	7.43	41.00	42.40	10.38	40.00	0.469
LDL-Cholesterol	106.86	38.63	100.00	112.93	41.86	112.00	117.43	38.49	112.00	0.506
TC/HDL-Cholesterol	4.33	1.35	3.95	4.63	1.14	4.34	5.13	2.24	4.78	0.089
Estradiol	9.76	7.61	7.45	11.96	9.80	8.20	11.50	10.11	7.00	0.853
IL-17	58.15	35.14	51.88	48.99	26.84	46.63	52.60	29.00	45.38	0.673
high sensitivity CRP	2.61	1.35	2.00	2.97	1.74	3.00	3.13	2.47	3.00	0.635

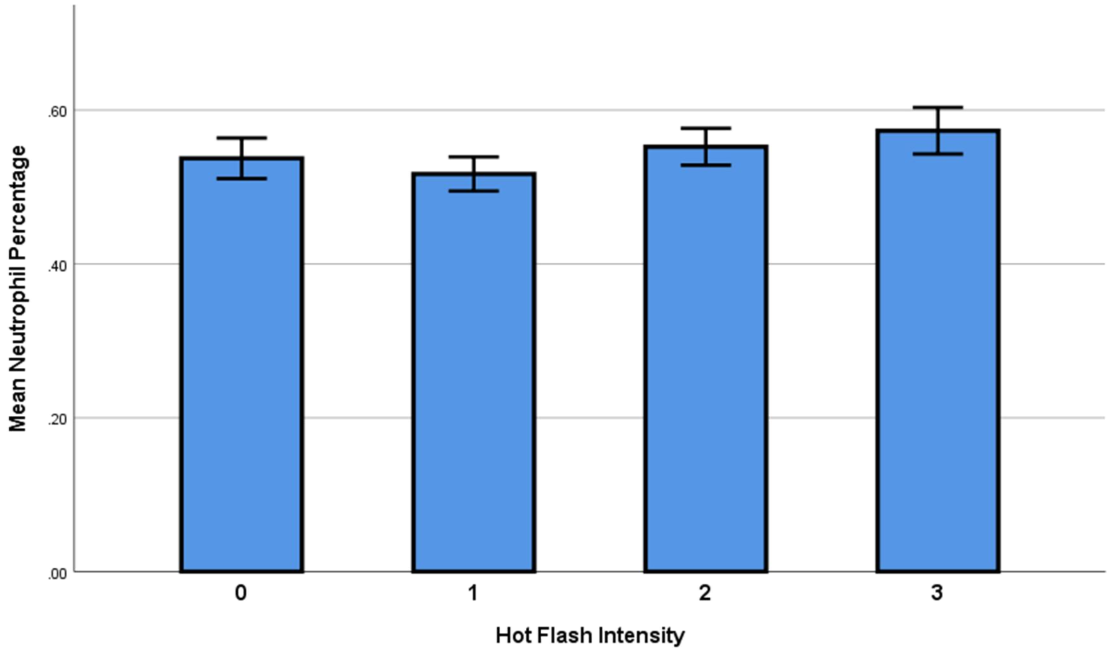

Sample 1-Sample 2	Test Statistic	Std. Error	Std. Test Statistic	Sig.	Adj. Sig.[a]
1-0	14.331	10.216	1.403	.161	.964
1-2	-22.910	10.032	-2.284	.022	.134
1-3	-33.788	10.583	-3.193	.001	.008
0-2	-8.579	10.158	-.845	.398	1.000
0-3	-19.457	10.703	-1.818	.069	.414
2-3	-10.878	10.527	-1.033	.301	1.000

Each row tests the null hypothesis that the Sample 1 and Sample 2 distributions are the same.

Asymptotic significances (2-sided tests) are displayed. The significance level is .05.

a. Significance values have been adjusted by the Bonferroni correction for multiple tests.

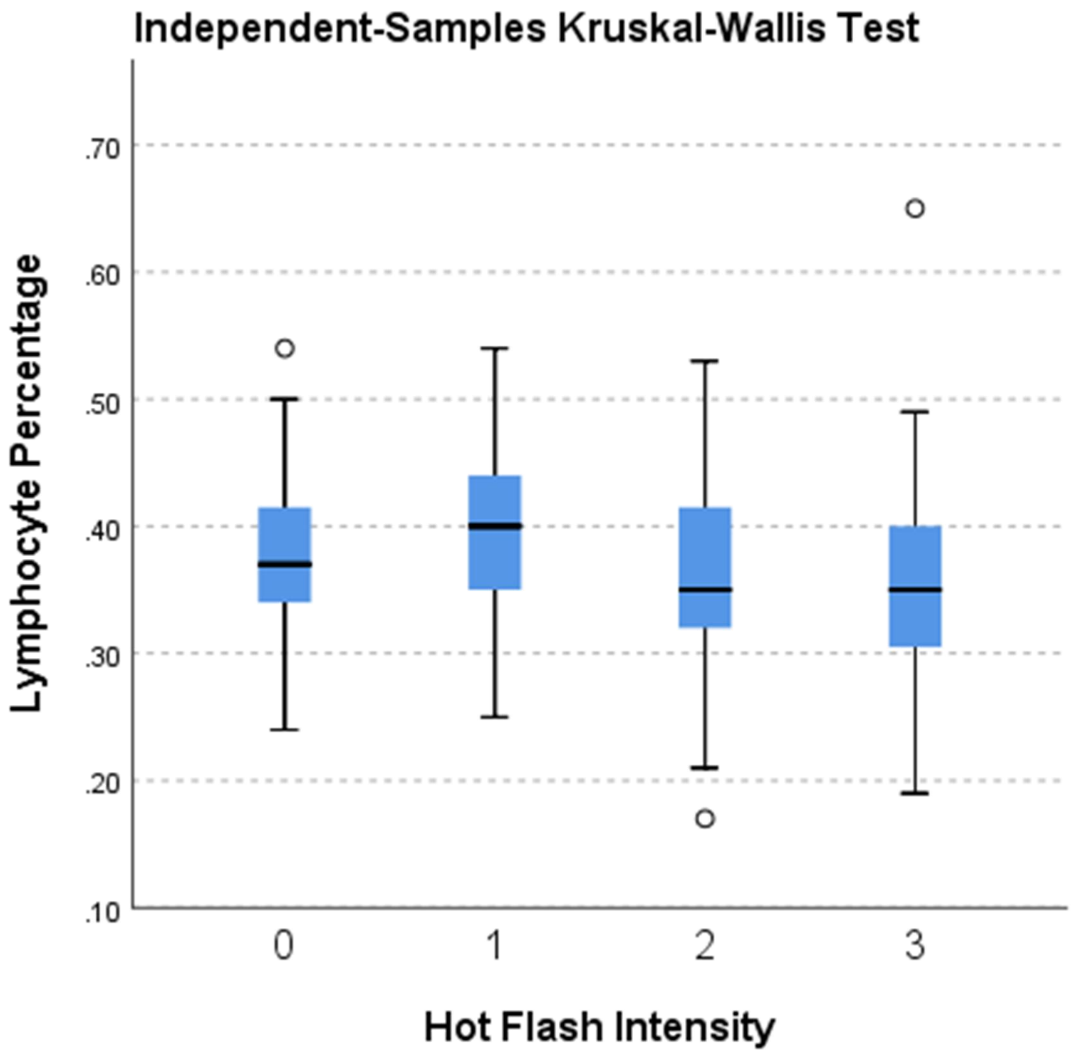

Sample 1-Sample 2	Test Statistic	Std. Error	Std. Test Statistic	Sig.	Adj. Sig.[a]
3-2	4.577	10.528	.435	.664	1.000
3-0	14.429	10.704	1.348	.178	1.000
3-1	28.157	10.584	2.660	.008	.047
2-0	9.851	10.159	.970	.332	1.000
2-1	23.580	10.033	2.350	.019	.113
0-1	-13.729	10.217	-1.344	.179	1.000

Each row tests the null hypothesis that the Sample 1 and Sample 2 distributions are the same.

Asymptotic significances (2-sided tests) are displayed. The significance level is .05.

a. Significance values have been adjusted by the Bonferroni correction for multiple tests.

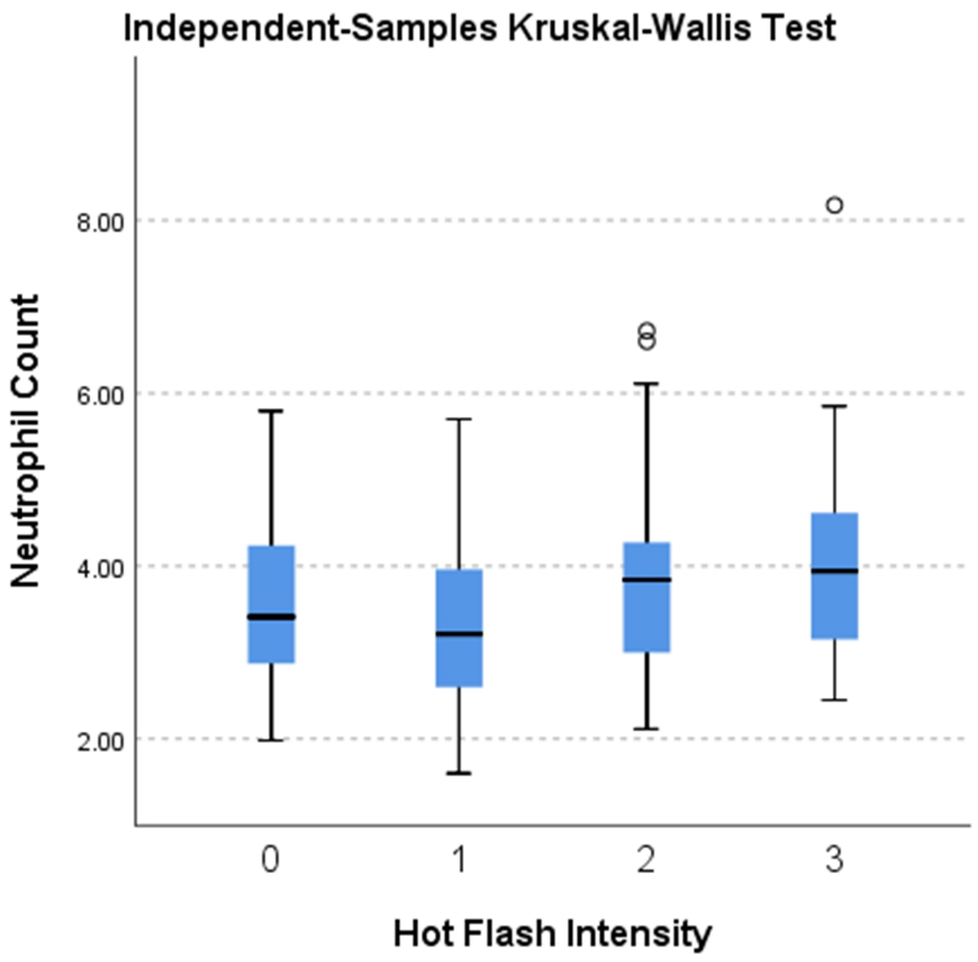

Sample 1-Sample 2	Test Statistic	Std. Error	Std. Test Statistic	Sig.	Adj. Sig.[a]
1-0	14.564	10.236	1.423	.155	.929
1-2	-22.534	10.051	-2.242	.025	.150
1-3	-30.936	10.604	-2.917	.004	.021
0-2	-7.970	10.178	-.783	.434	1.000
0-3	-16.371	10.724	-1.527	.127	.761
2-3	-8.402	10.548	-.797	.426	1.000

Each row tests the null hypothesis that the Sample 1 and Sample 2 distributions are the same.

Asymptotic significances (2-sided tests) are displayed. The significance level is .05.

a. Significance values have been adjusted by the Bonferroni correction for multiple tests.

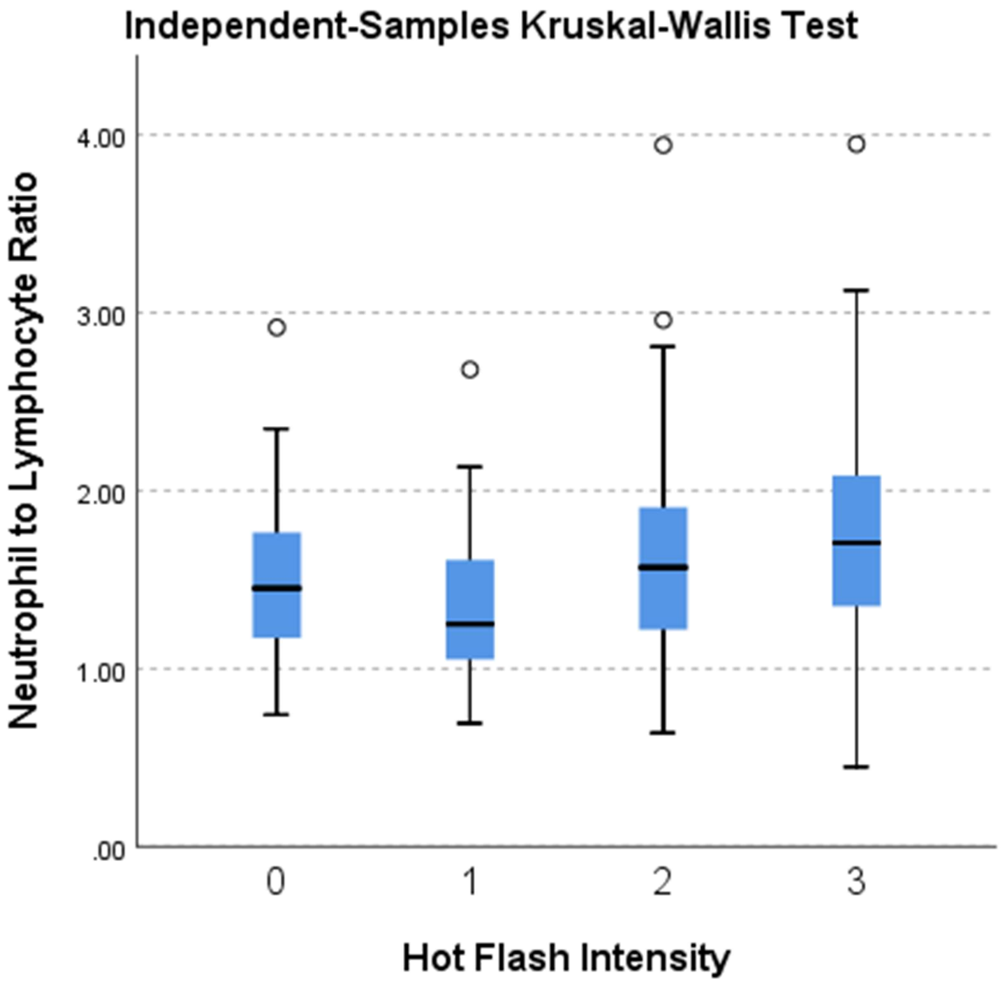

Sample 1-Sample 2	Test Statistic	Std. Error	Std. Test Statistic	Sig.	Adj. Sig.[a]
1-0	14.072	10.235	1.375	.169	1.000
1-2	-25.461	10.051	-2.533	.011	.068
1-3	-31.767	10.603	-2.996	.003	.016
0-2	-11.389	10.177	-1.119	.263	1.000
0-3	-17.695	10.723	-1.650	.099	.593
2-3	-6.306	10.547	-.598	.550	1.000

Each row tests the null hypothesis that the Sample 1 and Sample 2 distributions are the same.

Asymptotic significances (2-sided tests) are displayed. The significance level is .05.

a. Significance values have been adjusted by the Bonferroni correction for multiple tests.

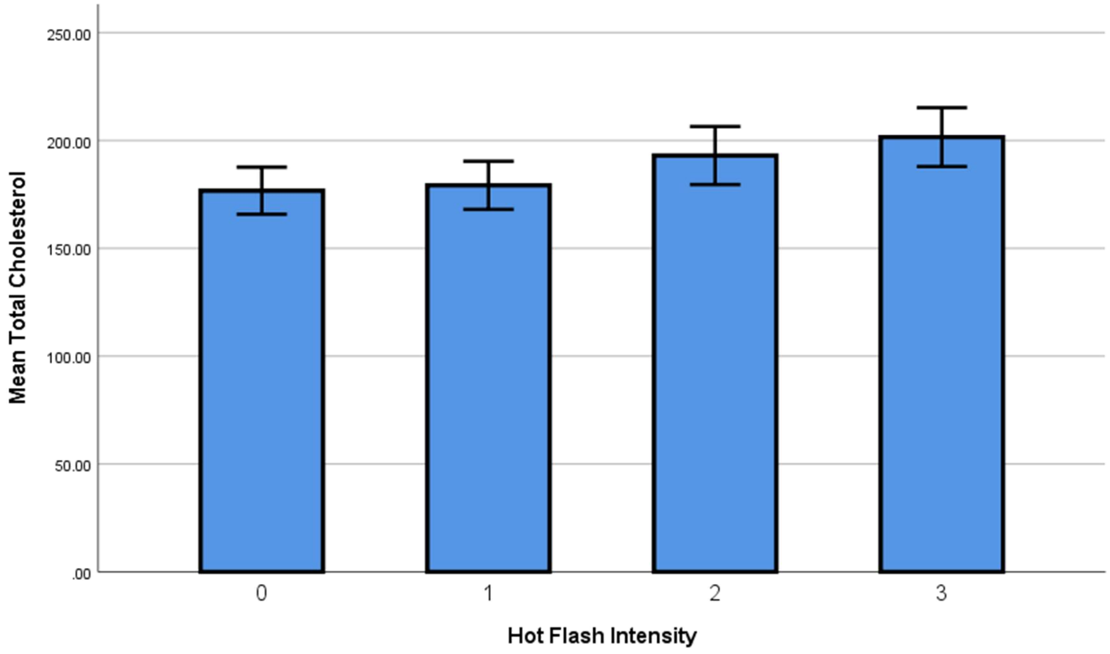

Pairwise Comparisons of Hot Flashes Intensity

Sample 1-Sample 2	Test Statistic	Std. Error	Std. Test Statistic	Sig.	Adj. Sig.[a]
0-1	-1.088	10.235	-.106	.915	1.000
0-2	-16.809	10.177	-1.652	.099	.592
0-3	-26.500	10.723	-2.471	.013	.081
1-2	-15.721	10.051	-1.564	.118	.707
1-3	-25.412	10.603	-2.397	.017	.099
2-3	-9.691	10.547	-.919	.358	1.000

Each row tests the null hypothesis that the Sample 1 and Sample 2 distributions are the same.

Asymptotic significances (2-sided tests) are displayed. The significance level is .05.

a. Significance values have been adjusted by the Bonferroni correction for multiple tests.

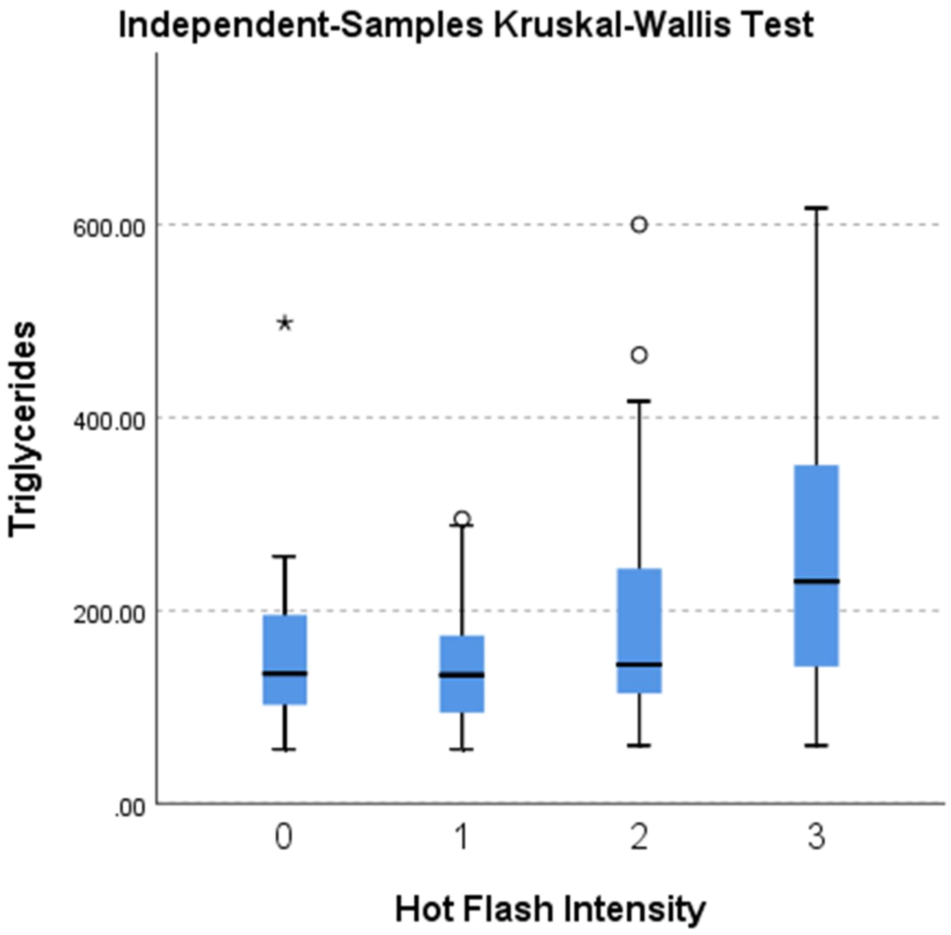

Pairwise Comparisons of Hot Flashes Intensity

Sample 1-Sample 2	Test Statistic	Std. Error	Std. Test Statistic	Sig.	Adj. Sig.[a]
1-0	5.267	10.236	.515	.607	1.000
1-2	-19.888	10.051	-1.979	.048	.287
1-3	-45.738	10.604	-4.313	.000	.000
0-2	-14.621	10.177	-1.437	.151	.905
0-3	-40.471	10.723	-3.774	.000	.001
2-3	-25.850	10.547	-2.451	.014	.085

Each row tests the null hypothesis that the Sample 1 and Sample 2 distributions are the same.
Asymptotic significances (2-sided tests) are displayed. The significance level is .05.
a. Significance values have been adjusted by the Bonferroni correction for multiple tests.

Variables in the Equation

		B	S.E.	Sig.	Exp(B)	95% C.I.for EXP(B)	
						Lower	Upper
Step 1[a]	Age	.622	.699	.374	1.862	.473	7.330
	Marriage Duration	-.039	.029	.182	.961	.908	1.019
	Menopausal Age	-.694	.699	.321	.500	.127	1.966
	Elapsed time since the last menstruation	-.053	.058	.361	.948	.846	1.063
	Lymphocyte to Monocyte Ratio	.046	.040	.254	1.047	.967	1.134
	Neutrophil to Lymphocyte Ratio	.556	.397	.162	1.743	.801	3.795
	Total Cholesterol	.004	.006	.489	1.004	.993	1.015
	Triglycerides	.004	.002	.066	1.004	1.000	1.009
	HDL-Cholesterol	.040	.028	.147	1.041	.986	1.100
	Constant	1.673	3.532	.636	5.330		
Step 9[a]	Marriage Duration	-.046	.024	.055	.955	.911	1.001
	Triglycerides	.004	.002	.040	1.004	1.000	1.009
	Constant	1.865	.893	.037	6.455		

a. Variable(s) entered on step 1: Age, Marriage Duration , Menopausal Age, Elapsed time since the last menstruation, Lymphocyte to Monocyte Ratio, Neutrophil to Lymphocyte Ratio, Total Cholesterol, Triglycerides, HDL-Cholesterol.

Parameter Estimates

Parameter	B	Std. Error	P-Value	Exp(B)	95% Wald Confidence Interval for Exp(B)	
					Lower	Upper
WC/HC(>85 / <=85)	1.064	.4760	.025	2.898	1.140	7.366
Menopausal Age	-.163	.0449	.000	.849	.778	.927
Elapsed time since the last menstruation	-.015	.0035	.000	.985	.978	.992
White Blood Cells	.207	.1052	.049	1.231	1.001	1.512
Monocyte Count	-3.122	1.2489	.012	.044	.004	.510
Neutrophil to Lymphocyte Ratio	.779	.2759	.005	2.180	1.270	3.744
Triglycerides	.009	.0016	.000	1.009	1.005	1.012
HDL-Cholesterol	.037	.0190	.048	1.038	1.000	1.077

Area Under the Curve

Test Result Variable(s)	Area	Std. Error[a]	Asymptotic Sig.[b]	Asymptotic 95% Confidence Interval	
				Lower Bound	Upper Bound
Triglycerides	.694	.048	.000	.600	.788
Neutrophil to Lymphocyte Ratio	.676	.050	.002	.578	.773

The test result variable(s): Triglycerides, Neutrophil to Lymphocyte Ratio has at least one tie between the positive actual state group and the negative actual state group. Statistics may be biased.

a. Under the nonparametric assumption

b. Null hypothesis: true area = 0.5

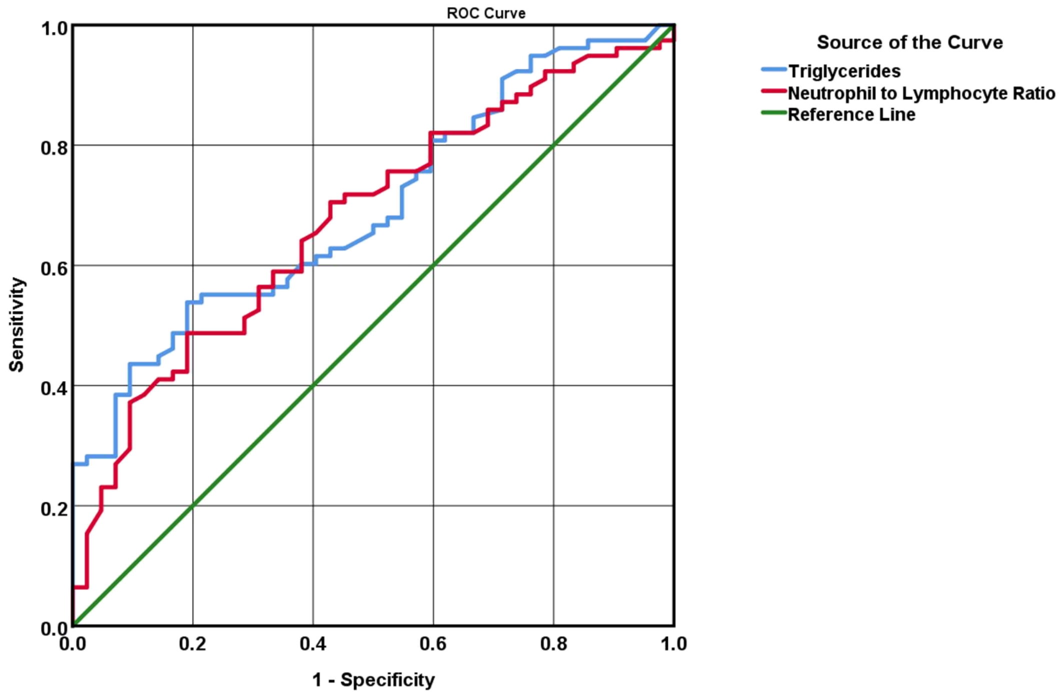

Area Under the Curve

Test Result Variable(s)	Area	Std. Error[a]	Asymptotic Sig.[b]	Asymptotic 95% Confidence Interval Lower Bound	Asymptotic 95% Confidence Interval Upper Bound
Triglycerides	.595	.049	.072	.499	.691
Neutrophil to Lymphocyte Ratio	.527	.052	.607	.425	.629

The test result variable(s): Triglycerides, Neutrophil to Lymphocyte Ratio has at least one tie between the positive actual state group and the negative actual state group. Statistics may be biased.

a. Under the nonparametric assumption

b. Null hypothesis: true area = 0.5

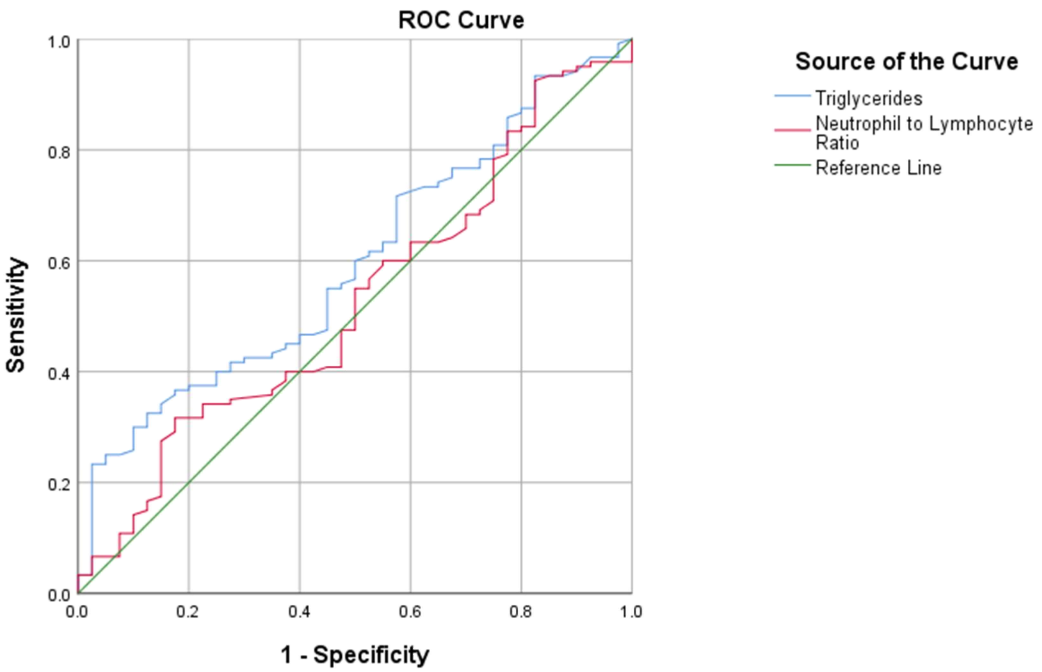

Diagonal segments are produced by ties.

Area Under the Curve

Test Result Variable(s)	Area	Std. Error[a]	Asymptotic Sig.[b]	Asymptotic 95% Confidence Interval Lower Bound	Asymptotic 95% Confidence Interval Upper Bound
Triglycerides	.681	.042	.000	.598	.763
Neutrophil to Lymphocyte Ratio	.634	.044	.003	.548	.720

The test result variable(s): Triglycerides, Neutrophil to Lymphocyte Ratio has at least one tie between the positive actual state group and the negative actual state group. Statistics may be biased.

a. Under the nonparametric assumption

b. Null hypothesis: true area = 0.5

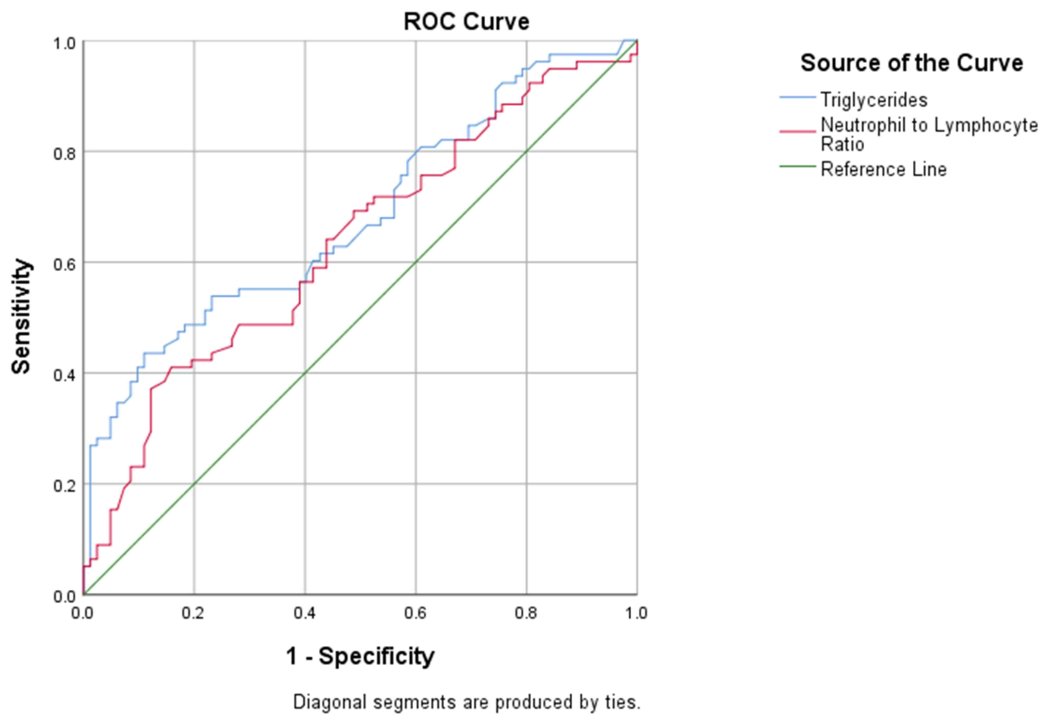

Area Under the Curve

Test Result Variable(s)	Area	Std. Error[a]	Asymptotic Sig.[b]	Asymptotic 95% Confidence Interval Lower Bound	Asymptotic 95% Confidence Interval Upper Bound
Triglycerides	.733	.053	.000	.629	.836
Neutrophil to Lymphocyte Ratio	.616	.055	.037	.509	.723

The test result variable(s): Triglycerides, Neutrophil to Lymphocyte Ratio has at least one tie between the positive actual state group and the negative actual state group. Statistics may be biased.

a. Under the nonparametric assumption

b. Null hypothesis: true area = 0.5

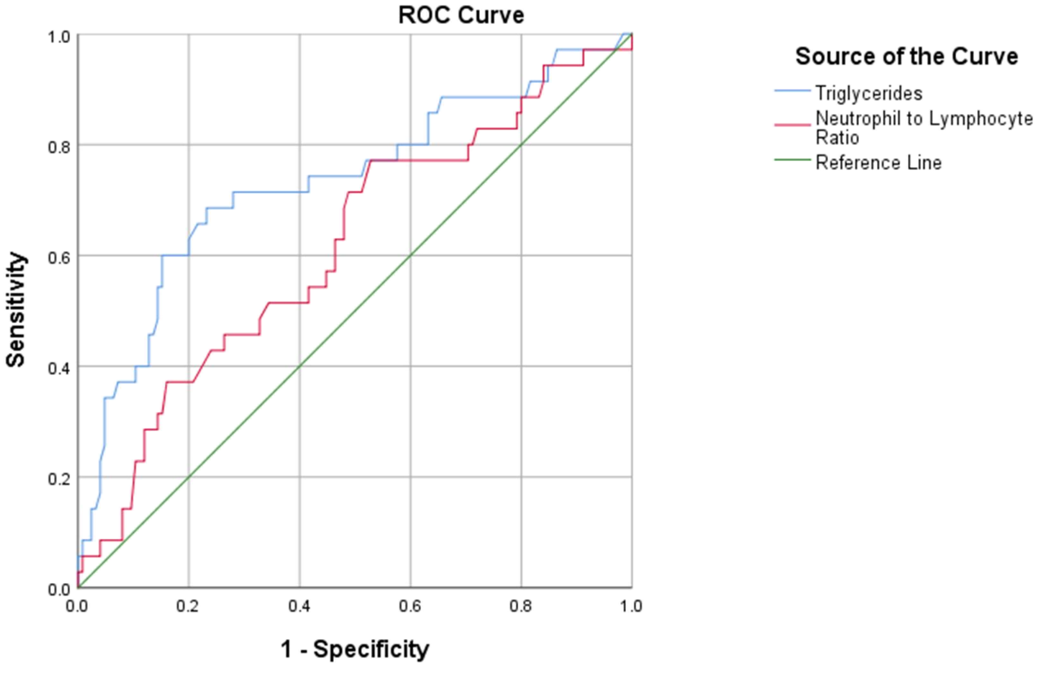

Diagonal segments are produced by ties.

Correlations							
			Total Cholesterol	Triglycerides	HDL-Cholesterol	LDL-Cholesterol	TC/HDL-Cholesterol
Spearman's rho	White Blood Cells	Correlation Coefficient	-.034	.162	-.155	-.141	.026
		Sig. (2-tailed)	.711	.077	.090	.124	.774
		N	120	120	120	120	120
	Neutrophil Percentage	Correlation Coefficient	-.111	-.162	-.085	.009	-.076
		Sig. (2-tailed)	.228	.077	.354	.924	.408
		N	120	120	120	120	120
	Lymphocyte Percentage	Correlation Coefficient	.105	.161	.060	-.007	.085
		Sig. (2-tailed)	.253	.080	.512	.938	.355
		N	120	120	120	120	120
	Monocyte Percentage	Correlation Coefficient	-.020	-.001	.119	-.064	-.083
		Sig. (2-tailed)	.829	.991	.197	.489	.370
		N	120	120	120	120	120
	Neutrophil Count	Correlation Coefficient	-.076	.046	-.154	-.102	-.020
		Sig. (2-tailed)	.407	.617	.093	.268	.829
		N	120	120	120	120	120
	Lymphocyte Count	Correlation Coefficient	.069	.243	-.097	-.096	.116
		Sig. (2-tailed)	.451	.007	.294	.296	.209
		N	120	120	120	120	120
	Monocyte Count	Correlation Coefficient	-.056	.095	.032	-.164	-.073
		Sig. (2-tailed)	.542	.300	.728	.074	.425
		N	120	120	120	120	120
	Neutrophil to Lymphocyte Ratio	Correlation Coefficient	-.119	-.156	-.073	.003	-.092
		Sig. (2-tailed)	.195	.088	.428	.978	.319
		N	120	120	120	120	120
	Lymphocyte to Monocyte Ratio	Correlation Coefficient	.039	.048	-.062	.034	.088
		Sig. (2-tailed)	.675	.599	.502	.709	.341
		N	120	120	120	120	120
	high sensitivity CRP	Correlation Coefficient	-.119	-.189	.164	-.148	-.268
		Sig. (2-tailed)	.194	.039	.074	.107	.003
		N	120	120	120	120	120

	IL-17	Correlation Coefficient	.038	.141	-.110	-.123	.074
		Sig. (2-tailed)	.677	.125	.230	.181	.423
		N	120	120	120	120	120
