# Supplementary material for: Interleukin-17, C-reactive protein, Neutrophil-to-Lymphocyte ratio, Lymphocyte-to-Monocyte ratio, and lipid profiles in healthy menopausal women with or without hot flashes: A cross-sectional study
Source: PLoS One. 2023 Nov 22;18(11):e0291804. doi: 10.1371/journal.pone.0291804 (PMC10664956; doi:10.1371/journal.pone.0291804)
Supplement: Attachment — Submitted filename: Response to Reviewers.docx [file pone.0291804.s005.docx]

**March 14, 2023**

**Dear Dr. Emily Chenette**

**Editor-in-Chief**

**PLOS ONE**

**Research article**

Interleukin-17, C-Reactive Protein, Neutrophil-to-Lymphocyte ratio, Lymphocyte-to-Monocyte ratio, and lipid profiles in healthy menopausal women with or without hot flashes: A cross-sectional study (PONE-D-22-24749)

Nazila Didevar, Parvaneh Rezasoltani, Arash Pourgholaminejad, Ehsan Kazemnezhad Leyli, Tahereh Seyednoori, Ziba Zahiri Sorouri

Thank you for your email (Jan 30, 2023) about our joint manuscript cited above. We appreciate your reviewers' valuable remarks and suggestions that have improved our manuscript. The revisions applied are as follows.

I value your kind and quick response.

Yours’ Sincerely,

**Parvaneh Rezasoltani, PhD**

**Corresponding Author**

Assistant Professor, Department of Midwifery, School of Nursing and Midwifery, Guilan University of Medical Sciences, Rasht, Iran

rezasoltani@gums.ac.ir; [rezasoltani49@gmail.com](mailto:rezasoltani49@gmail.com)

**Editors’ comments**

**Academic Editor**

*-* *The authors are encouraged to write more clearly so that an international readership can understand the main issues addressed in this important paper.*

**Authors’ reply**: With respect and gratitude, we edited the article appropriately to clarify the content.

*-* ***Materials and Methods -*** *Page 7; Lines 149 – 153: The authors wrote, “Based on Fig 1 a total of 1987 women were* ***interviewed****, and 1816 of them were excluded from the study in terms of the lack of inclusion criteria. There were 171 eligible women, 11 of whom were unwilling to continue the cooperation, and a total of 160 were recruited. According to their response to HFs status, participating women were divided into two groups without HFs (N꞊40) and with HFs (N꞊120).” It is unclear what kind of interview was conducted on the initial sample of 1987.*

**Authors’ reply**: In this study, we interviewed all the menopausal women individually and asked them the same questions in a cosecutive manner, according to the inclusion criteria.

*- I think it flows better if the authors state that the women did not meet the inclusion criteria, rather than state that they lacked the inclusion criteria.*

**Authors’ reply**: We appreciate your recommendation. Therefore, concerning your two latter suggestions, we altered the above section to “Based on Fig 1, the first author (ND) individually interviewed a total of 1987 women in a consecutive manner, according to the inclusion criteria. As the sampling proceeded, she excluded 1816 women from the study because they did not meet the inclusion criteria. Finally, she collected 171 eligible women, out of which 11 were unwilling to continue the cooperation. Therefore, a total of 160 women were enrolled in the study. According to their response to HFs status, the participating women were divided into two groups without HFs (N꞊40) and with HFs (N꞊120).” (Page 7; Lines 153-159)

*- It is quite interesting that the process of selection of the eligible sample led to the exact number of women calculated as the minimum sample size. Is this approach reproducible by other researchers? Did the authors stop recruiting the moment they attained the minimum sample size, or did recruitment continue until the end of that work day? What steps did the researchers put in place to avoid selection bias?*

**Authors’ reply**: We value your consideration. We emphasize that menopausal women with different statuses might have visited the hospital on various days. We recruited them on definite days randomly using consecutive sampling. In other words, we enlisted eligible menopausal women who visited the hospital randomly only based on meeting the inclusion criteria. Thus, the researchers had no role in their selection. Therefore, other researchers will be able to apply such a method, but due to the randomness of the women included in the study, they may obtain different results. Furthermore, we mention that we continued recruiting women until the end of the workday we achieved the total sample size. Moreover, we note that we used a consecutive method for sampling. Therefore, we could replace the excluded women with others as the sampling proceeded. In this way, we attained the estimated sample size. Thus, we revised the related part as: “Finally, she collected 171 eligible women, out of which 11 were unwilling to continue the cooperation. Therefore, a total of 160 women were enrolled in the study.” (Page 7; Lines 156-157)

*- Page 7; line 154 – 155: The authors wrote, “...were given a card to record their HFs number for two weeks in a pilot manner.” Authors should edit to read, “... were given a card to record the number of HFs they experienced over a two-week period as part of a pilot study.”*

**Authors’ reply**: Thank you very much for your advice. We revised the sentence as you suggested: “ The women with HFs, who had experienced HFs during the three months before enrollment, were given a card and asked to record the number of HFs they experienced over a two-week period as part of a pilot study.” (Page 7; Lines 160-161)

*-* ***Results*** *- Figure 1: The authors state that 1816 women were excluded in the first stage but only 1716 women were accounted for. Why were the other 100 women excluded? These 100 women need to be reflected in Figure 1.*

**Authors’ reply**: With respect and expressions of regret, the mentioned point was only a typographical mistake. Reconsidering the data, we noticed that the number of women excluded from the study due to diabetes was 241, of which 141 were mistyped. We have corrected the mentioned point with many thanks for your consideration (Page 8; Fig. 1).

***- Discussion*** *- This section needs to be more concisely written as it is currently difficult to read and understand. Are there any recommendations for clinical practice, based on the findings of this study?*

**Authors’ reply**: We would appreciate informing you that we rewrote the discussion section and made it more concise. We also added a few suggestions for a clinical approach in this regard.

**Reviewer #1**

*- Lines 43-46: The following statement in the Abstract section should be written more clearly since not all those markers were measured in serum samples and even more, some of them were calculated: „The serum levels of Interleukin-17 (IL-17), C-Reactive Protein (CRP), Neutrophil-to-Lymphocyte Ratio (NLR), Lymphocyte-to-Monocyte Ratio (LMR), Total Cholesterol (TC), Triglycerides (TG), Low-Density Lipoprotein Cholesterol (LDL-C), High-Density Lipoprotein Cholesterol (HDL-C), and TC/HDL-C were measured in each group“.*

**Authors’ reply**: With many thanks and respect, we inform you that based on your comment, we rewrote and modified the mentioned point as follows: “In addition to clinical variables and HFs experience, we measured the fasting serum levels of SIMs and lipid profiles (LPs), including Interleukin-17 (IL-17), high-sensitivity C-Reactive Protein (hs-CRP), Total Cholesterol (TC), Triglycerides (TG), Low-Density Lipoprotein Cholesterol (LDL-C), and High-Density Lipoprotein Cholesterol (HDL-C) in each group. Then, we calculated TC/HDL-C concerning the related variables and determined Neutrophil-to-Lymphocyte Ratio (NLR), and Lymphocyte-to-Monocyte Ratio (LMR), according to Complete Blood Count (CBC) quantitative parameters in each group.” (Page 2; Lines 43-50)

*- Did the Authors exclude women with CRP ≥10 mg/L in order to exclude acute inflammation?*

**Authors’ reply**: Many thanks for your notice. Referring to our data, we observed that there were only two menopausal women (one in each severity value of 1 and 2) who had hs-CRP levels≥10 mg/L. None of them had clinical symptoms of inflammation. Moreover, they stated no disease history to put them in the exclusion criteria.

*- In the Statistical analysis subsection it should be mentioned that continuous data with non-normal distribution were presented as median (IQ range).*

**Authors’ reply**: With gratitude for your consideration, we included the suggested item in such a way: “The continuous variables with normal distribution were presented as the mean [standard deviation (SD)], those with non-normal distribution as the median (IQ range), and the categorical variables as frequency (percentage).” (Page 11; Lines 250-252)

*- The same results should be presented either in tables or in figures, but not both (e.g. Table 4 and Figures 3, 4).*

**Authors’ reply**: Thank you very much for your recommendation. We revised the content based on your comment (Page 13; Line 290 / Page 14; Line 309 / Page 15; Line 344 / Page 16; Line 349).

*- Why did not the Authors include IL-17 in the correlation analysis (Table 7)?*

**Authors’ reply**: With many thanks for your consideration, we declare that the mentioned point was a typographical mistake. We added the missing IL-17 to the correlation table, as below: (Page 21, Table 7)

| **TC/HDL-C** | **LDL-C** | **HDL-C** | **TG** | **TC** |  | |
| --- | --- | --- | --- | --- | --- | --- |
| 0.074 | -0.123 | -0.110 | 0.141 | 0.038 | **Correlation coefficient** | **IL-17** |
| 0.423 | 0.181 | 0.230 | 0.125 | 0.677 | **Sig. (2-tailed)** |  |
| 120 | 120 | 120 | 120 | 120 | **N** |  |

*- The Discussion section should be shortened in order to be more clear, coherent and concise.*

**Authors’ reply**: We would appreciate the opportunity to implement your advice. Therefore, we rewrote and modified the discussion section to be clear and more concise.

*- Lines 470-471: the Authors are not cited properly (i.e. reference 23): „Moreover, Pourgholaminejad et al. reported an increase in the neutrophil count with an increase in IL-17 [23]“.*

**Authors’ reply**: Below, we appreciatively corrected the citation: “Tahmasebinia et al. also reported raised IL-17 levels associated with increased neutrophil counts [23].” (Page 24; Lines 480-481)

*- Lines 488-490: the Authors are not cited properly (i.e. reference 19): „Belkadi et al. observed a raise in TC, LDL-C and TC/HDL-C and a drop in HDL-C in menopausal women and attributed these alterations to estradiol deficiency in menopausal women [19]“.*

**Authors’ reply**: Many thanks for your attentive remark. We adjusted the citation as follows: “TC, LDL-C, and TC/HDL-C levels increased, and HDL-C levels diminished in menopausal women, as indicated by Taleb-Belkadi et al. These alterations were ascribed to estradiol deficiency in menopause [19].” (Page 25; Lines 501-503)

*- Line 534: the following statement should be written to be more clear: „Study samples may not have reported their exact HFs number“.*

**Authors’ reply**: Based on your careful remark, we corrected this sentence to be more intelligible: “Participants recorded the number of their daily HFs subjectively. Accordingly, they may not have registered their accurate number of HFs.” (Page 27; Lines 552-553)

*- Line 536: the following statement should be written to be more clear: „HFs had a relationship with TG“.*

**Authors’ reply**: We gratefully revised this sentence to be more comprehensible, according to your comment: “Concisely, we found that TG was related to HFs in menopausal women. Indeed, menopausal women with HFs had higher levels of TG than those without HFs.” (Page 27; Lines 555-556)

*- The units are missing in some figures.*

**Authors’ reply**: We state that we corrected all missing units in the titles of the figures with special thanks for your carefulness (Page 21; Lines 419 and 422 / Page 22; Lines 425, 428, 432, and 435).

**Reviewer #2**

*- Please explain all abbreviations in the abstract and manuscript.*

**Authors’ reply**: According to your comment, we explained all abbreviations in both the abstract and manuscript.

***Abstract***

*1) Abstract should be informative, I think abstract need to be rewrite to be more easy to get!*

**Authors’ reply**: With thanks and respect, we rewrote the abstract to be more intelligible (Pages 2-3).

*2) Keywords: are these keywords are Mesh terms? Word that serves as a keyword, as to the meaning of that condition must be a Mesh term.*

**Authors’ reply**: Much obliged for your attentive comment; we state that all the keywords are MeSH terms. While designing the proposal for this study, we checked the keywords through PubMed and MeSH databases according to headings.

***Introduction***

*- There are some sentences that are difficult to understand, and the paper needs an English reviewer. Please edit.*

*- introduction is too long I think it would be possible to summarize some parts.*

**Authors’ reply**: Thank you very much for your notice. According to your comment, we edited this part.

***Methods***

*- I couldn’t find any methodological tools and definition for evaluation the food intake in these patients as authors know nutritional intake and status play a very important role in post-menopausal women’s health and it should be evaluated as a very critical confounder in this relationship.*

**Authors’ reply**: We thankfully mention that we did not use any methodological tools to assess nutrition. Instead, we asked some questions about nutritional status in a questionnaire, the validity and reliability of which were verified by ten faculty members of the School of Nursing and Midwifery of Guilan Medical Sciences. These questions were as shown: “Do you ever consume fatty and fast food? If so, how often do you use it?” Therefore, according to your suggestion, we put the non-use of tools in the analysis of nutritional status in the limitations so that researchers in the future can use the related tools to evaluate the exact effects of nutrition in this study (Page 27; Lines 548-551).

*- What about physical activity, I also couldn’t find in the method part any explanation for evaluation of physical activity of participants.*

**Authors’ reply**: As I said about nutrition, we did not use a methodological tool for physical activities either. We asked valid and reliable questions verified by ten faculty members of the School of Nursing and Midwifery of Guilan University of Medical Sciences. These questions (in the demographic and reproductive form) were as follows: “Do you ever work out? If so, what kind of activity, how many times a week, and how long (in minutes) each time?” (Pages 17-18; Tables 1 and 2).

***Results***

*- Evaluation of inflammatory markers especially Interleukins and CRP are definitely related to the time of a day, what is the exact time of sampling and are there any planned and schedule for this?*

**Authors’ reply**: All participants had to take a blood test between 8 and 10 am, concerning the laboratory regulations. Therefore, we planned a schedule according to which participants could refer to the research laboratory and take the blood test (Page 9; Line 210).

***Discussion***

*- The strength and limitations of the study should be extensively described. For example limited number of sample size and also do not evaluating the very important confounders such as food intake and physical activity should be described as limitation.*

**Authors’ reply**: Thank you very much for your consideration. Based on your comments, we explained this part more extensively (Page 27; Lines 544-551).
