# Supplementary material for: Interleukin-17, C-reactive protein, Neutrophil-to-Lymphocyte ratio, Lymphocyte-to-Monocyte ratio, and lipid profiles in healthy menopausal women with or without hot flashes: A cross-sectional study
Source: PLoS One. 2023 Nov 22;18(11):e0291804. doi: 10.1371/journal.pone.0291804 (PMC10664956; doi:10.1371/journal.pone.0291804)
Supplement: Attachment — Submitted filename: Response to Reviewers.docx [file pone.0291804.s006.docx]

**July 27, 2023**

**Dear Dr. Emily Chenette, PhD**

**Editor in Chief, PLOS ONE**

**Research article**

Interleukin-17, C-Reactive Protein, Neutrophil-to-Lymphocyte ratio, Lymphocyte-to-Monocyte ratio, and lipid profiles in healthy menopausal women with or without hot flashes: A cross-sectional study (PONE-D-22-24749R1)

Nazila Didevar, Parvaneh Rezasoltani, Arash Pourgholaminejad, Ehsan Kazemnezhad Leyli, Tahereh Seyednoori, Ziba Zahiri Sorouri

Thank you for your email (July 5, 2023) about our joint manuscript cited above. We appreciate your reviewers' valuable remarks and suggestions that have improved our manuscript. The revisions applied are as follows.

I value your kind and quick response.

Sincerely yours

**Parvaneh Rezasoltani, PhD**

**Corresponding Author**

Assistant Professor, Department of Midwifery, School of Nursing and Midwifery, Guilan University of Medical Sciences, Rasht, Iran

rezasoltani@gums.ac.ir; [rezasoltani49@gmail.com](mailto:rezasoltani49@gmail.com)

**Editors’ comments**

**Reviewer #2**

*- All the comments has been fully addressed and the revision was made, and I was satisfied with the response.*

**Authors’ reply**: We gratefully express our special thanks for your consideration and valuable comments, which helped a lot our manuscript to be improved.

**Reviewer #3**

*- The paper is well revised with a clear language. The topic is important for the region where the study took place. Before proceed authors should briefly discuss recent studies PMID: 30744450 PMID: 28472898*

**Authors’ reply**: We would appreciate the opportunity to implement your guidance. Therefore, we briefly cited the related key features of the mentioned papers in the appropriate sections:

- “Furthermore, CRP levels were not associated with total menopause rating scale (MRS) symptom score, Kaya et al. realized [29].” (Page 23; Lines 468-469)

- “Besides, Kaya et al. revealed that elevated TG levels were associated with higher total MRS symptom scores. However, they did not observe any significant difference in symptom severity-based groups regarding HDL-C levels [29]. Similarly, increased levels of TG were associated with higher total MRS scores in menopausal women, Cengiz et al. noticed [31].” (Pages 25-26; Lines 516-521)

**Journal Requirements**:

*Please review your reference list to ensure that it is complete and correct. If you have cited papers that have been retracted, please include the rationale for doing so in the manuscript text, or remove these references and replace them with relevant current references. Any changes to the reference list should be mentioned in the rebuttal letter that accompanies your revised manuscript. If you need to cite a retracted article, indicate the article’s retracted status in the References list and also include a citation and full reference for the retraction notice.*

**Authors’ reply**: With many thanks, we let you know that we rechecked the list of references and made sure they were correct and accurate. Moreover, we state that no retracted papers were among those we used. Also, if available, we added their PMID to the list of references and wrote down the full title of the journals in citations. We also mention that according to the suggestion of one of the respected reviewers, we added two papers to the previous list of references, which include numbers 29 and 31. The updated list is as follows (Pages 30-34; Lines 618- 762):

**References**

1. Shifren JL. Schiff I. Menopause. In: Berek JS. Berek & Novak’s Gynecology Essentials: Wolters Kluwer Health; 2020. p. 934.

2. Casper RF. Clinical Manifestations and Diagnosis of Menopause; 2023 [cited 2023 Jun 29]. Database: UpToDate [Internet]. Available from: https://www.uptodate.com/contents/clinical-manifestations-and-diagnosis-of-menopause

3. Santen RJ, Loprinzi CL, Casper RF. Menopausal Hot Flashes; 2023 [cited 2020 Apr 27]. Database: UpToDate [Internet]. Available from: https://www.uptodate.com/contents/menopausal-hot-flashes

4. Thurston RC, Joffe H. Vasomotor symptoms and menopause: findings from the Study of Women's Health across the Nation. Obstetrics and Gynecology Clinics of North America. 2011;38(3):489-501. doi: 10.1016/j.ogc.2011.05.006 PMID: 21961716

5. Franco OH, Muka T, Colpani V, Kunutsor S, Chowdhury S, Chowdhury R, et al. Vasomotor symptoms in women and cardiovascular risk markers: Systematic review and meta-analysis. Maturitas. 2015;81(3):353-361. doi: 10.1016/j.maturitas.2015.04.016 PMID: 26022385

6. Thurston RC, El Khoudary SR, Sutton-Tyrrell K, Crandall CJ, Gold EB, Sternfeld B, et al. Vasomotor symptoms and lipid profiles in women transitioning through menopause. Obstetrics and Gynecology. 2012;119(4):753-761. doi: 10.1097/AOG.0b013e31824a09ec PMID: 22433339

7. Tuomikoski P, Mikkola TS, Hämäläinen E, Tikkanen MJ, Turpeinen U, Ylikorkala O. Biochemical markers for cardiovascular disease in recently postmenopausal women with or without hot flashes. Menopause. 2010;17(1)145-151.doi: 10.1097/gme.0b013e3181acefd5 PMID: 19602991

8. Muka T, Imo D, Jaspers L, Colpani V, Chaker L, van der Lee SJ, et al. The global impact of non-communicable diseases on healthcare spending and national income: a systematic review. European Journal of Epidemiology. 2015;30(4):251-277. doi: 10.1007/s10654-014-9984-2 PMID: 25595318

9. Muka T. Women' s health: Implications of diet and cardiometabolic risk factors. Doctoral dissertation, the Netherlands, the Erasmus University Rotterdam. 2016. Available from: <https://repub.eur.nl/pub/93261/>

10. Huang WY, Hsin IL, Chen DR, Chang CC, Kor CT, Chen TY, et al. Circulating interleukin-8 and tumor necrosis factor-alpha are associated with hot flashes in healthy postmenopausal women. PLoS One. 2017;12(8):e0184011. doi: 10.1371/journal.pone.0184011 PMID: 28846735

11. Chedraui P, Jaramillo W, Pérez-López FR, Escobar GS, Morocho N, Hidalgo L. Pro-inflammatory cytokine levels in postmenopausal women with the metabolic syndrome. Gynecological Endocrinology. 2011;27(9):685-691. doi: 10.3109/09513590.2010.521270 PMID: 20937002

12. Karaoulanis SE, Daponte A, Rizouli KA, Rizoulis AA, Lialios GA, Theodoridou CT, et al. The role of cytokines and hot flashes in perimenopausal depression. Annals of General Psychiatry. 2012;11:9. doi: 10.1186/1744-859X-11-9 PMID: 22490187

13. Thurston RC, El Khoudary SR, Sutton-Tyrrell K, Crandall CJ, Gold E, Sternfeld B, et al. Are vasomotor symptoms associated with alterations in hemostatic and inflammatory markers? Findings from the Study of Women's Health Across the Nation. Menopause. 2011;18(10):1044-1051. doi: 10.1097/gme.0b013e31821f5d39 PMID: 21926929

14. Malutan AM, Dan M, Nicolae C, Carmen M. Proinflammatory and anti- inflammatory cytokine changes related to menopause. Menopause review/Przegląd Menopauzalny. 2014;13(3):162-168. doi: 10.5114/pm.2014.43818 PMID: 26327849

15. Miossec P, Korn T, Kuchroo VK. Interleukin-17 and type 17 helper T cells. The New England Journal of Medicine. 2009;361(9):888-898. doi: 10.1056/NEJMra0707449 PMID: 19710487

16. Molnár I, Bohaty I, Somogyiné-Vári É. High prevalence of increased interleukin-17A serum levels in postmenopausal estrogen deficiency. Menopause. 2014;21(7):749- 752. doi: 10.1097/GME.0000000000000125 PMID: 24253487

17. Waliullah S, Sharma V, Srivastava RN, Pradeep Y, Mahdi AA, Kumar S. IL-17A and IL-23 cytokines and their relation with estrogen in post menopausal osteoporosis. International Journal of Orthopaedics Sciences. 2018;4(1):864-866. doi: 10.22271/ortho.2018.v4.i1m.124

18. Joffe HV, Ridker PM, Manson JE, Cook NR, Buring JE, Rexrode KM. Sex hormone-binding globulin and serum testosterone are inversely associated with C-reactive protein levels in postmenopausal women at high risk for cardiovascular disease. Annals of Epidemiology. 2006;16(2):105-112. doi: 10.1016/j.annepidem.2005.07.055 PMID: 16216530

19. Taleb-Belkadi O, Chaib H, Zemour L, Fatah A, Chafi B, Mekki K. Lipid profile, inflammation, and oxidative status in peri- and postmenopausal women. Gynecological Endocrinology. 2016;32(12):982-985. doi: 10.1080/09513590.2016.1214257 PMID: 27558905

20. Chen Y, Zhang Y, Zhao G, Chen C, Yang P, Ye S, et al. Difference in leukocyte composition between women before and after menopausal age, and distinct sexual dimorphism. PLoS One. 2016;11(9):e0162953. doi: 10.1371/journal.pone.0162953 PMID: 27657912

21. Pourgholaminejad A, Tahmasebinia F. The role of Th17 cells in immunopathogenesis of neuroinflammatory disorders. Neuroimmune Diseases: Springer. 2019. p. 83-107. doi: 10.1007/978-3-030-19515-1_3

22. Flannigan KL, Ngo VL, Geem D, Harusato A, Hirota SA, Parkos CA, et al. IL-17A-mediated neutrophil recruitment limits expansion of segmented filamentous bacteria. Mucosal Immunology. 2017;10(3):673-684. doi: 10.1038/mi.2016.80 PMID: 27624780

23. Tahmasebinia F,Pourgholaminejad A. The role of Th17 cells in auto-inflammatory neurological disorders. Progress in Neuropsychopharmacology and Biological Psychiatry. 2017;79(Pt B):408-416. doi: 10.1016/j.pnpbp.2017.07.023 PMID: 28760387

24. Lee JS, Kim NY, Na SH, Youn YH, Shin CS. Reference values of neutrophil-lymphocyte ratio, lymphocyte-monocyte ratio, platelet-lymphocyte ratio, and mean platelet volume in healthy adults in South Korea. Medicine. 2018;97(26):e11138. doi: 10.1097/MD.0000000000011138 PMID: 29952958

25. Harlow SD, Gass M, Hall JE, Lobo R, Maki P, Rebar RW, et al. Executive summary of the Stages of Reproductive Aging Workshop + 10: addressing the unfinished agenda of staging reproductive aging. Menopause. 2012;19(4):387-395. doi: 10.1097/gme.0b013e31824d8f40 PMID: 22344196

26. Tao M, Shao H, Li C, Teng Y. Correlation between the modified Kupperman Index and the Menopause Rating Scale in Chinese women. Patient Preference and Adherence. 2013;7:223-229. doi: 10.2147/PPA.S42852 PMID: 23569361

27. Gast G-CM, Samsioe GN, Grobbee DE, Nilsson PM, van der Schouw YT. Vasomotor symptoms, estradiol levels and cardiovascular risk profile in women. Maturitas. 2010;66(3):285-290. doi: 10.1016/j.maturitas.2010.03.015 PMID: 20400247

28. Gaffen SL. The role of interleukin-17 in the pathogenesis of rheumatoid arthritis. Current Rheumatology Reports. 2009;11(5):365-370. doi: 10.1007/s11926-009-0052-y PMID: 19772832

29. Kaya C, Cengiz H, Yeşil A, Ekin M, Yaşar L. The relation among steroid hormone levels, lipid profile and menopausal symptom severity. Journal of Psychosomatic Obstetrics & Gynecology. 2017;38(4):284-291. doi: 10.1080/0167482X.2017.1321633 PMID: 28472898

30. Davison S, Davis SR. New markers for cardiovascular disease risk in women: impact of endogenous estrogen status and exogenous postmenopausal hormone therapy. The Journal of Clinical Endocrinology & Metabolism. 2003;88(6):2470 -2478. doi: 10.1210/jc.2002-021929 PMID: 12788842

31. Cengiz H, Kaya C, Suzen Caypinar S, Alay I. The relationship between menopausal symptoms and metabolic syndrome in postmenopausal women. Journal of Obstetrics and Gynaecology. 2019;39(4):529-533. doi:10.1080/01443615.2018.1534812 PMID: 30744450

32. Song M-S, Yoo Y-K, Choi C-H, Kim N-C. Effects of nordic walking on body composition, muscle strength, and lipid profile in elderly women. Asian Nursing Research. 2013;7(1):1-7. doi: 10.1016/j.anr.2012.11.001 PMID: 25031209

*- While revising your submission, please upload your figure files to the Preflight Analysis and Conversion Engine (PACE) digital diagnostic tool.  PACE helps ensure that figures meet PLOS requirements.*

**Authors’ reply**: We have appreciatively uploaded all our figure files to the PACE digital diagnostic tool, where they are accessible through the archive tab.
